# Factors Associated with Willingness to Work with Older Adults Among Healthcare Professionals and Students: A Cross-Sectional Study in a Polycrisis Context

**DOI:** 10.3390/healthcare14213584

**Published:** 2026-10-23

**Authors:** Rudy S. Younes, Mohamad Alayan, Lynn Barq, Mariam Allaw, Rim Assaf, Ferial Rajha, Mirna Abboud Mzawak

**Affiliations:** 1 Department of Psychology and Social Sciences, Faculty of Arts and Sciences, Holy Spirit University of Kaslik (USEK), Jounieh P.O. Box 446, Lebanon; rudy.younes@usek.edu.lb (R.S.Y.); mohamad.h.alayan@net.usek.edu.lb (M.A.); 2 IDEES Multidisciplinary Research Group, Faculty of Arts and Sciences, Holy Spirit University of Kaslik (USEK), Jounieh P.O. Box 446, Lebanon; 3 Faculty of Public Health, Lebanese University, Badaro, Beirut P.O. Box 6573/14, Lebanon; lynn.barq.1@st.ul.edu.lb (L.B.); mariam.allaw@st.ul.edu.lb (M.A.); rim.assaf.1@st.ul.edu.lb (R.A.); ferial.rajha@ul.edu.lb (F.R.); 4 Department of Nursing, Faculty of Public Health, Lebanese German University (LGU), Jounieh P.O. Box 206, Lebanon

**Keywords:** geriatric care, healthcare workforce, older adults, ageism, financial satisfaction, intergenerational relationships, good health and well-being

## Abstract

**Background/Objectives:** The growing demand for care of older persons is occurring alongside challenges in attracting healthcare professionals to work with older adults. In Lebanon, these challenges are particularly relevant amid prolonged economic and healthcare crises and a rapidly aging population. Avoidance of working in the care of older persons may further contribute to workforce shortages and limit the availability of age-appropriate healthcare. This study examined factors associated with willingness to work with older adults, focusing on financial satisfaction, ageism, perceived efficacy of education and training, close relationships with older adults, and knowledge about aging. **Methods:** A cross-sectional study was conducted among 209 healthcare professionals and students. **Results:** Financial satisfaction was positively associated with willingness to work with older adults. Having close relationships with several older adults was also associated with greater willingness. No statistically significant associations were observed for ageism, knowledge about aging, or perceived efficacy of education and training. **Conclusions:** Willingness to work with older adults may be shaped more by financial and relational factors than by general ageist attitudes. These findings highlight the need for workforce strategies that address both economic conditions and meaningful exposure to older adults. Improving financial conditions and opportunities for sustained intergenerational contact may help strengthen interest in the care of older persons.

## 1. Introduction

The number of people aged 65 years and older worldwide is expected to double by 2050, exceeding 1.6 billion individuals [1]. This demographic shift is increasing the demand for healthcare systems to provide care that is responsive to the needs of older populations. Aging is also associated with a greater prevalence of multimorbidity, chronic disease, and complex healthcare needs, requiring sustained management and often more comprehensive clinical care [2].

Addressing these changes therefore requires greater investment in healthcare that is competent in the care of older persons and an adequately prepared workforce. Lebanon illustrates these challenges clearly. More than 10% of the Lebanese population is already aged over 65 years, representing the highest proportion in the Arab region, while life expectancy at birth is approximately 76 years [3]. This demographic transition is further affected by the continued emigration of younger adults, reducing the proportion of working-age individuals available to support a growing older population [4,5]. As the population ages, healthcare professionals are increasingly required to manage multimorbidity, chronic conditions, and complex and potentially prolonged care needs [6].

Lebanon’s healthcare system, however, has limited capacity to respond to these changing needs [7,8]. The country has a documented shortage of personnel trained in the care of older persons, limited insurance coverage for older adults, no functioning national pension system, and no comprehensive national policy on aging [9]. Moreover, community-based support programs for older adults remain limited in Lebanon, including services addressing psychological well-being, while the range of available care facilities is relatively narrow. The healthcare system continues to rely substantially on nursing home structures, which may limit the diversity of settings in which older adults can receive care and in which healthcare professionals can work with them [10].

These longstanding structural limitations have been further exacerbated by the polycrisis that Lebanon has experienced since 2019 [11], including economic collapse, the banking crisis, the Beirut port explosion, the COVID-19 pandemic, and prolonged political instability [12]. These overlapping crises have contributed to the emigration of healthcare workers, had repercussions on the well-being of healthcare workers, placed additional financial pressure on healthcare institutions, and weakened families’ ability to provide informal care for older relatives [13,14]. The broader consequences of these conditions may also extend to perceptions and treatment of older adults [15]. Thus, Lebanon faces increasing demand for care of older persons alongside constraints in both the availability of specialized personnel and the quality of care provided to older adults [9].

The challenge of attracting healthcare professionals to older adults’ care is not unique to Lebanon. A systematic review found that medical students generally preferred younger patients and acute, potentially curable conditions, while the perceived complexity of caring for older patients was an important factor discouraging interest in the care of older persons [16]. The same review suggested that clinical exposure to older patients was more consistently associated with increased interest in careers caring for older persons than classroom-based teaching alone. Similarly, nursing students have been found to show limited interest in the care of older persons despite receiving education related to aging [17]. These findings suggest that the presence of content on the care of older persons within health education does not necessarily translate into willingness to pursue work with older adults.

Several factors may contribute to this willingness [16,17,18,19]. One potential factor is financial circumstances. In contexts where healthcare professionals face substantial economic pressure, career choices may be influenced by perceived financial returns and the relative attractiveness of different specialties. Haron et al. [17], for example, identified financial considerations among factors relevant to nursing students’ attitudes toward the care of older persons. This may be particularly relevant in Lebanon, where the economic crisis has substantially affected healthcare professionals and institutions. A second factor concerns education and training. Although clinical exposure to older adults has been associated with greater interest in careers caring for older persons [16], the amount or design of classroom-based education has not consistently predicted willingness to work in the care of older persons [17]. Perceived preparedness may therefore be more relevant than the mere presence of content on the care of older persons, although this relationship remains insufficiently understood.

A third potential factor is ageism. Ageist attitudes toward older adults have been documented among healthcare professionals and students across Arab countries, particularly in low- and middle-income settings, with evidence also demonstrating the presence of ageism among Lebanese healthcare workers [15,20]. Internationally, a systematic review found that lower knowledge about aging and less clinical experience with older adults were associated with higher levels of ageism among healthcare workers [21]. Ageism may therefore influence willingness to work with older adults by shaping how healthcare professionals perceive aging and older patients [18,19,20]. However, negative attitudes toward older adults may not necessarily translate into avoidance of work with older persons, particularly when professional decisions are also influenced by economic, educational, and contextual considerations.

Other factors may operate as protective influences. Knowledge about aging has been associated with more favorable attitudes toward older adults [22,23,24]. For example, a study among nursing students in Türkiye identified inaccurate beliefs about aging alongside generally positive but domain-specific attitudes toward older adults [25]. Consistent with this, the systematic review by Fernández-Puerta et al. [21] found that greater knowledge about aging was associated with lower ageism across several studies.

Close relationships with older adults may reduce psychological distance through interpersonal contact and familiarity [24,26]. This is consistent with intergroup contact theory, according to which meaningful contact between social groups can reduce negative attitudes toward the other group [27,28]. Among healthcare workers and students, intergenerational contact and a personal desire to work with older adults have been identified among the factors most consistently associated with lower ageism [20,21]. Importantly, the quality of contact may be more relevant than its frequency alone [29]. Relationships with older adults may therefore be relevant not only to attitudes toward aging but also to willingness to work with older adults.

Despite these proposed explanations, the factors associated with willingness to work with older adults, as distinct from general attitudes toward older adults, remain insufficiently studied in Lebanon. This distinction is important because factors associated with ageism may not necessarily be the same factors that influence professional willingness to work with older adults. For example, previous research has shown that training content does not necessarily predict career willingness, even when training is expected to influence attitudes toward the care of older persons [17]. Identifying the factors associated with willingness may therefore provide a more direct basis for workforce planning, educational interventions, and strategies to increase interest in the care of older persons [18], particularly in a healthcare system facing both demographic and economic pressures.

The objective of the present study was therefore to examine sociodemographic, financial, and psychosocial factors associated with willingness to work with older adults among healthcare professionals and students in Lebanon. Specifically, the study examined financial satisfaction, ageism, perceived efficacy of education and training, relational closeness to older adults, and knowledge about aging as potential factors associated with willingness to work with older adults. Based on the existing literature, five hypotheses were proposed:

**H1.**  *Greater financial satisfaction will be associated with greater willingness to work with older adults.*

**H2.**  *Higher ageism will be associated with lower willingness to work with older adults.*

**H3.**  *Greater perceived efficacy of one’s education and training, reflecting greater perceived preparedness to work with older adults, will be associated with greater willingness to work with older adults.*

**H4.**  *Close relationships with older adults will be associated with greater willingness to work with older adults.*

**H5.**  *Greater knowledge about aging will be associated with greater willingness to work with older adults.*

## 2. Materials and Methods

### 2.1. Study Design and Participants

A cross-sectional study [30] was conducted using a convenience sample of individuals with varying educational and professional backgrounds related to older adults. Participants were recruited through an online survey distributed using convenience sampling techniques. Eligibility criteria included being aged 18 years or older and providing informed consent to participate. The full description of the sample is provided in the Supplementary Materials. Since the study is cross-sectional, it adhered to the STROBE Statement guidelines (see Supplementary Materials).

A minimum sample size [31] was calculated using G*Power software version 3.1.97, based on a linear multiple regression model with R^2^ deviation from zero. Assuming a theoretical effect size of f^2^ = 0.15 (medium effect), an alpha error probability of 0.05, a beta error probability of 0.05 (95% power), and a maximum of 11 covariates in the final model, the minimum required sample size was 178 participants. The final sample consisted of 209 participants, exceeding the required minimum.

### 2.2. Data Collection

Data were collected through an online questionnaire available in both English and Arabic, similar to previous studies [32,33], since they are two commonly used languages in Lebanon. The questionnaire was distributed through several WhatsApp groups using convenience sampling [34]. The questionnaire collected information on participants’ sociodemographic and contextual characteristics, ageist attitudes, knowledge about aging, and financial satisfaction.

Participants provided information on age, sex, education level, employment status, years of education in elder-related fields, professional experience, current work with older adults, perceived efficacy of their education or training in preparing them to work with older adults, completion of courses on aging or older adults, having a close relationship with an older person outside the family, and having older family members. Perceived efficacy of education or training was assessed using a single-item question: “In your opinion, was the training you received sufficient to work with older people?” Responses were recorded on a 5-point Likert scale ranging from 1 (Not at all) to 5 (Completely), with higher scores indicating greater perceived sufficiency of training to work with older people. Scales were used to measure key study variables.

**Willingness to Work with Older Adults:** Willingness to work with older adults was assessed using a single item, inspired by a previous study focusing on this construct [18]: “To what extent are you willing to work with older people (e.g., in a nursing home or geriatric care)?” Participants responded on a five-point Likert scale ranging from 1 (strongly unwilling) to 5 (strongly willing), with higher scores indicating greater willingness to work with older adults. A single-item measure was used because the study aimed to capture participants’ overall willingness to work with older adults, rather than to assess its potentially distinct dimensions. Moreover, to our knowledge, no reliable multi-item scales are available to measure this variable. Although multi-item scales can provide broader coverage of complex constructs, single-item measures may be appropriate when the target construct is relatively concrete and can be expressed through a clear, direct question [35,36]. Previous methodological research has also demonstrated that single-item measures can show acceptable reliability and validity under appropriate conditions [35,36]. The use of a single item was therefore considered suitable for the study’s specific objective, while recognizing that it does not permit an assessment of internal consistency.

**Ageism:** Ageism was assessed using the Arabic version of the Kogan Attitudes Toward Older People Scale [37]. For the purposes of the present study, the negative-attitudes subscale was used to specifically assess negative attitudes toward older adults. Higher scores indicate greater ageist attitudes. The use of a single subscale has been adopted in previous studies focusing specifically on negative attitudes toward older adults [38]. In the present study, the negative-attitudes subscale demonstrated good internal consistency, with a Cronbach’s alpha of 0.875 and McDonald’s omega of 0.878.

**Financial Satisfaction:** Financial satisfaction was assessed using a single-item measure adapted from the Wellcome Global Monitor Mental Health Survey 2020. Participants were asked, “How do you feel about your current financial situation?” Responses were recorded on a five-point Likert scale ranging from “Not satisfied at all” to “Very satisfied”, with higher scores indicating greater financial satisfaction. This item was selected because the Wellcome Global Monitor Mental Health Survey 2020 has been tested and adapted across multiple sociocultural contexts, including Lebanon [39].

**Knowledge About Aging:** Knowledge about aging was assessed using the Facts on Aging Quiz I, a 25-item true/false instrument measuring factual knowledge about aging and older adulthood [40]. Each item was scored according to whether the response was correct, with one point awarded for a correct answer and zero points for an incorrect answer. The total score ranged from 0 to 25, with higher scores indicating greater knowledge about aging. The Facts on Aging Quiz I has been widely used to assess knowledge about aging [41,42]. For cultural appropriateness, one item from the original instrument (“Most older adults have no desire or capacity for sexual relations.”) was replaced with an item from the Facts on Aging Quiz II (“More older persons have chronic illnesses that limit their activity than do younger persons.”) [43]. This modification was made because topics related to sexuality are not commonly discussed publicly in Lebanon [44].

The complete questionnaire was administered in both English and Arabic. The ageism measure had previously been validated and was available in both languages; therefore, no additional translation was required. The remaining questionnaire items were translated by two bilingual researchers and reviewed by a third bilingual researcher. As the Facts on Aging Quiz was available only in English, it was translated into Arabic using a forward–backward translation procedure [45]. First, a bilingual researcher translated the questionnaire items from English into Arabic. The Arabic version was then independently translated back into English by another bilingual researcher to identify potential discrepancies in meaning between the original and translated versions. The translations were subsequently reviewed independently by four bilingual researchers to ensure that the items retained their intended meaning. Finally, the questionnaire was pilot tested with 10 individuals, who were asked to provide feedback on any items they found unclear or difficult to understand. All items were reported to be clear, and no changes were made following pilot testing. The questionnaire was then distributed to participants.

### 2.3. Data Analysis

Data were analyzed using JASP version 0.19.3.0. Descriptive statistics, including frequencies, percentages, means, and standard deviations, were calculated to summarize participant characteristics and study variables. The normality of the continuous variables was assessed using skewness and kurtosis. Values between −2 and +2 were considered indicative of an acceptable normal distribution [46,47].

Independent-samples t-tests and one-way analyses of variance (ANOVAs) were conducted to examine differences in willingness scores across sociodemographic and contextual variables. Effect sizes were calculated using Cohen’s d for independent-samples t-tests, and omega squared (ω^2^) for ANOVAs. Where the assumption of homogeneity of variances was not met, the corresponding non-parametric test was used. Specifically, the Mann–Whitney U test was used as an alternative to the independent-samples *t*-test where appropriate, with the rank-biserial correlation reported as the corresponding effect size. Pearson correlation analyses were performed to assess associations between age, perceived efficacy of education and training, financial satisfaction, ageism, and willingness to work with older adults [48].

Subsequently, a multiple linear regression analysis was planned to identify factors independently associated with willingness to work with older adults. However, the linearity assumption was not met based on the inspection of residual and scatter plots (see Supplementary Materials). As a result, an ordinal logistic regression analysis was conducted instead using IBM’s Statistical Package for the Social Sciences (SPSS) version 27 to identify factors independently associated with willingness to work with older adults. In line with methodological guidelines and previous studies, factors that showed a *p* < 0.25 in the bivariate analyses were considered for inclusion in the regression model, using purposeful selection of variables at the screening stage to avoid excluding potentially important covariates [49,50,51,52]. The *p* < 0.25 criterion was used only as a screening rule for candidate covariates and not as a formal criterion for statistical significance in the final interpretation of the regression results. Statistical significance was considered at a *p*-value < 0.05.

## 3. Results

Table 1 presents the sociodemographic and contextual characteristics of the participants. The sample comprised 209 individuals, predominantly female (74.6%). The majority held a bachelor’s degree (48.8%), with an additional 16.7% holding a master’s degree. Most participants were students (55.5%), followed by employed individuals (35.4%). Nearly half had more than three years of education in an elder-related field (49.8%), and 40.2% had 0–1 year of professional experience. The majority reported currently working with older adults (72.2%), and most had taken courses on aging or older adults (65.1%). Most participants reported a close relationship with an older person outside the family (78.5%, mostly with several older individuals), and the large majority reported having older family members (89.0%).

Table 2 presents the descriptive statistics for the main study variables. Participants had a mean age of 28.56 years (SD = 12.01). The mean willingness score was 3.11 (SD = 0.97) on a five-point scale, and perceived efficacy averaged 2.17 (SD = 0.86). The mean ageism score was 52.32 (SD = 13.98), knowledge about aging averaged 12.74 (SD = 2.77), and financial satisfaction had a mean of 3.02 (SD = 1.07). At the item level, responses to the willingness question were distributed as follows: 6.7% selected 1 (strongly unwilling), 15.8% selected 2, 43.1% selected 3, 28.7% selected 4, and 5.7% selected 5 (strongly willing), indicating that most participants reported moderate willingness to work with older adults.

Table 3 presents the bivariate analyses examining associations between sociodemographic and contextual variables and willingness scores. Having a close relationship with an older person outside the family was the only variable significantly associated with willingness (*p* = 0.002, ω^2^ = 0.051), with participants who had a close relationship with several older people reporting the highest mean willingness (M = 3.30 ± 0.92), compared to those without such a relationship (M = 2.76 ± 0.91) or with only one older person (M = 2.89 ± 1.05). Sex, education level, employment status, years of elder-related education, work experience, current work with older adults, having taken courses on aging, and having older family members did not reach statistical significance.

Table 4 presents the Pearson correlations among the study variables. Financial satisfaction showed a significant, weak positive correlation with willingness (r = 0.202, *p* = 0.003), indicating that greater financial satisfaction was associated with greater willingness to work with older adults. Age was significantly correlated with efficacy (r = 0.163, *p* = 0.019). Efficacy, knowledge, and ageism were not significantly correlated with willingness.

Following these bivariate analyses, a multiple linear regression analysis was planned to identify factors independently associated with willingness to work with older adults. However, the linearity assumption was not met based on the inspection of residual and scatter plots (see Supplementary Materials). In addition, willingness was measured on a five-point ordinal scale. Thus, an ordinal logistic regression (proportional odds model with a logit link) was conducted instead.

Table 5 presents the ordinal regression model predicting willingness to work with older adults, accounting for efficacy, ageism, financial satisfaction, close relationship with an older person, and elder family members. The model fit significantly better than the intercept-only model (χ^2^(7) = 25.431, *p* = 0.001; Nagelkerke R^2^ = 0.123). Financial satisfaction was a significant positive predictor (B = 0.341, SE = 0.124, Wald χ^2^ = 7.556, *p* = 0.006; OR = 1.406, 95% CI [1.103, 1.793]). Each one-unit increase in financial satisfaction was associated with about 41% higher odds of reporting a higher level of willingness. Having a close relationship with many older people was also a significant positive predictor, relative to a close relationship with only one older person (B = 0.827, SE = 0.378, Wald χ^2^ = 4.793, *p* = 0.029; OR = 2.286, 95% CI [1.091, 4.792]). Efficacy, ageism, having no close relationship with an older person, and having elder family members did not reach statistical significance in the model. The test of parallel lines was marginally significant (χ^2^(21) = 33.318, *p* = 0.043), suggesting that the proportional odds assumption may not fully hold.

As a sensitivity analysis to support the regression model, the multiple linear regression model initially planned was also conducted and is presented in the Supplementary Materials. The findings were consistent with those of the ordinal logistic regression. Financial satisfaction (B = 0.150, SE = 0.061, *p* = 0.016) and having a close relationship with many older people (B = 0.481, SE = 0.189, *p* = 0.011) were again the only significant positive predictors of willingness. Efficacy, ageism, and having elder family members remained non-significant. This alignment suggests that the main findings are robust to the choice of analytic approach.

## 4. Discussion

This study examined sociodemographic, financial, and psychosocial factors associated with willingness to work with older adults among healthcare professionals and students in Lebanon. Specifically, it examined financial satisfaction, ageism, perceived efficacy of education and training, close relationships with older adults, and knowledge about aging as potential correlates of willingness. Financial satisfaction was positively associated with willingness in both the bivariate and multivariate analyses, while having close relationships with several non-family older adults was also associated with greater willingness. In contrast, no statistically significant associations were observed in the present sample between willingness and ageism, perceived efficacy of education and training, or knowledge about aging. Thus, of the five hypotheses, H1 was supported and H4 was partially supported, whereas H2, H3, and H5 were not supported.

### 4.1. Financial Satisfaction

First, financial satisfaction was positively associated with willingness to work with older adults, supporting H1. The association was present at the bivariate level and remained significant after accounting for efficacy, ageism, and relational factors. This finding is consistent with previous research suggesting that financial considerations can influence preferences for work with older persons [17]. The finding may be particularly relevant in Lebanon, where the economic crisis has substantially affected healthcare professionals and the healthcare sector [7,11]. When financial circumstances are uncertain, career decisions may be influenced by the perceived economic attractiveness of different specialties [53,54,55]. Care of older persons may therefore compete with specialties that are perceived to offer greater financial returns or professional opportunities. Importantly, financial satisfaction was not significantly associated with ageism in the present sample. This suggests that its association with willingness may be more closely related to professional or career considerations than to general attitudes toward older adults linked to financial circumstances. However, the cross-sectional design does not allow us to determine whether financial circumstances influence willingness or whether other factors related to career choice influence both financial satisfaction and willingness.

### 4.2. Relationships with Older Adults

Second, close relationships with older adults showed a more specific pattern. Participants who reported close relationships with several older people had greater willingness to work with older adults than those who reported no such relationship [20,26,27]. However, no statistically significant difference in willingness was observed for participants with only one close relationship, or for those with older family members. This provides partial support for H4 and is broadly consistent with literature suggesting that interpersonal contact with older adults can be associated with more favorable attitudes and greater interest in working with them [21,29]. This pattern may partly reflect the commonality of intergenerational relationships in Lebanon, where having at least one older family member is the norm [56,57]; consequently, the presence of a single relationship may be less useful in distinguishing participants, whereas having close relationships with several older adults may reflect broader exposure to older people. The finding that several relationships, rather than a single relationship, were associated with willingness may indicate that broader and more sustained exposure to older adults is more relevant than the simple presence of one relationship. However, this interpretation should be treated cautiously because the study did not assess the frequency, quality, or emotional closeness of these relationships.

The absence of a statistically significant association between having older family members and willingness may partly reflect the limited variability in this measure [58]. Nearly 90% of participants reported having older family members, leaving relatively few participants in the comparison group. This restricted variation makes it difficult to detect differences between those with and without older family members. Family relationships may also represent a different type of contact from voluntary relationships outside the family. Contact with older relatives can involve family responsibilities and caregiving demands [56,57], whereas non-family relationships may reflect more voluntary forms of intergenerational interaction [59,60]. The present data cannot establish whether these differences explain the pattern observed, but they suggest that simply having older relatives may not be equivalent to having meaningful interpersonal exposure that influences professional willingness.

### 4.3. Ageism and Knowledge About Aging

Third, no statistically significant association was observed between ageism and willingness to work with older adults in the present sample, either in the correlation analysis or in the regression model, and H2 was therefore not supported. Similarly, H5 was examined at the bivariate level, and no statistically significant correlation was observed between knowledge about aging and willingness (r = −0.020, *p* = 0.776); H5 was therefore not supported. Although knowledge about aging was significantly associated with ageism, this relationship did not translate into a significant association between knowledge and willingness. The non-significant association of ageism with willingness to work with older adults differs from the expectation that more negative attitudes toward older adults would be associated with less willingness to work with them [18,19]. However, the absence of a statistically significant association in the present sample does not imply that ageism or knowledge of aging is irrelevant to the care of older persons. Ageism and willingness to work with older adults represent related but distinct constructs. A person may hold negative beliefs about aging while still being willing to work with older adults because professional decisions are influenced by other considerations, including financial circumstances, career opportunities, and personal experiences [53,54,55]. One possible explanation is that, amid Lebanon’s prolonged and severe socioeconomic and political crises, immediate financial and career-related considerations may have become more influential in shaping willingness to work with older adults than age-related attitudes. In this context, financial satisfaction may be particularly salient, potentially outweighing the influence of ageism on professional preferences. This context-specific explanation may help account for the divergence from findings reported in other countries, although further research is needed to determine whether the relative influence of ageism and other factors varies across different socioeconomic and crisis contexts.

### 4.4. Perceived Efficacy of Education and Training

Fourth, no statistically significant association was observed between perceived efficacy of education and training and willingness, providing no support for H3. Participants who felt better prepared by their formal education or training did not report significantly greater willingness to work with older adults [61]. This finding is noteworthy given previous evidence that clinical exposure to older adults may increase interest in careers caring for older persons, while classroom-based education alone has shown less consistent effects [16,17]. One possible explanation is that perceived preparedness and willingness to choose work with older persons represent different stages of professional decision-making. Feeling adequately prepared may be more relevant to confidence and effectiveness after entering practice with older persons than to the initial decision to pursue such work [62,63].

### 4.5. Attitudes and Willingness to Work with Older Adults

The present findings therefore suggest that general attitudes toward older adults may not translate directly into professional willingness. This distinction is consistent with behavioral models such as the theory of planned behavior, in which attitudes represent one component of the process leading to behavioral intentions rather than being equivalent to the intention itself. Other factors like context can play a significant role as well [64,65,66]. In the present study, financial satisfaction was associated with willingness whereas no statistically significant association was observed for ageism, which provides some support for considering willingness as a distinct outcome rather than assuming that reducing ageism will necessarily increase interest in work with older persons. At the same time, because the study did not directly assess other components of behavioral intention, such as subjective norms or perceived behavioral control, these mechanisms cannot be tested directly here [67,68].

All in all, the findings point to a distinction between the factors associated with willingness to work with older adults and those associated with attitudes toward older adults. Financial satisfaction and having several close non-family relationships with older adults were associated with willingness, whereas no statistically significant associations were observed for ageism, knowledge about aging, or perceived efficacy of education and training. This does not mean that financial circumstances or interpersonal contact are the only determinants of willingness. Nevertheless, the findings suggest that increasing the workforce caring for older persons may require attention to practical and experiential factors in addition to efforts to improve attitudes or increase classroom-based education. In particular, interventions aimed at reducing ageism should not necessarily be assumed to increase willingness to work with older adults, and educational efforts should not be evaluated solely on the basis of whether participants feel better prepared. These may represent related but distinct areas of workforce development in the care of older persons.

### 4.6. Limitations

Several limitations should be considered when interpreting these findings. First, the cross-sectional design prevents conclusions about causality or temporal direction [69]. Although greater financial satisfaction was associated with greater willingness, for example, the study cannot determine whether financial circumstances influence willingness to pursue work with older persons or whether other characteristics related to career preferences influence both variables.

Second, participants were recruited using convenience sampling, which limits the representativeness of the sample [70]. The sample may overrepresent individuals who are already engaged in healthcare education or practice and may therefore differ from the broader Lebanese healthcare workforce. Furthermore, recruitment through WhatsApp networks may have introduced selection bias, as individuals who were connected to the researchers’ professional or academic networks, were more active on WhatsApp, or were more interested in the study topic, may have been more likely to encounter the survey and participate. This recruitment approach may also have excluded or underrepresented individuals with limited access to these networks or lower engagement with online surveys [71]. Consequently, participants may have differed from nonparticipants in their professional backgrounds, experiences with older adults, or willingness to work in the care of older persons, potentially influencing the observed associations. Although the sample size exceeded the estimated minimum required for the regression analysis, it should not be considered representative of healthcare professionals and students nationally.

In line with this, the sample was heavily weighted toward nursing participants, meaning that the findings may more accurately reflect the perspectives of individuals in nursing than those of other healthcare professionals and students. As a result, the findings may not be equally generalizable across healthcare professions, particularly those that differ in their training, professional responsibilities, and exposure to older adults. Future studies should aim to recruit more balanced samples across healthcare disciplines to better capture potential professional differences in willingness to work with older adults and to assess the extent to which the present findings apply to the broader healthcare workforce.

Third, willingness to work with older adults and financial satisfaction were each assessed using a single item. Single-item measures are practical and can constitute a reliable measurement tool [36,72], but they provide less information than multi-item measures and may not capture the different dimensions of willingness or financial well-being [72,73]. Future studies could use validated multi-item measures to provide a more comprehensive assessment of these constructs.

Fourth, all measures were self-reported, creating the possibility of social desirability bias [74,75]. This may be particularly relevant to ageism, as participants may be reluctant to report negative attitudes toward older adults. Such responses could reduce the observed variability in ageism and potentially weaken its association with willingness.

Fifth, the measures of interpersonal relationships captured the presence and number of relationships with older adults but did not assess their frequency, duration, quality, or emotional closeness. Consequently, the finding for non-family relationships cannot establish whether the number of relationships itself is relevant or whether participants with several relationships also experience more frequent or higher-quality contact [76]. Future research should assess the quality and nature of intergenerational contact more directly.

Finally, the regression model explained a relatively small proportion of the variance in willingness [77]. This indicates that important factors were not captured in the present analysis. Willingness to work in the care of older persons is likely shaped by a combination of individual preferences, professional expectations, and broader structural conditions that extend beyond the factors examined in this study. Future studies should consider additional influences on career preferences related to the care of older persons, including perceived professional status, expected career opportunities, workload, working conditions, salary expectations, organizational support, social norms, and the quality and duration of clinical exposure to older adults. Factors such as perceived emotional demands, concerns about burnout, opportunities for professional development, or personality traits may also influence whether individuals view the care of older persons as an attractive career path [78,79,80].

### 4.7. Healthcare Implications

The findings have several implications for workforce development in the care of older persons in Lebanon. First, the independent association between financial satisfaction and willingness suggests that economic considerations should be taken into account when developing strategies to attract healthcare professionals to the care of older persons. Financial incentives, improved remuneration, scholarships for geriatric specialization, loan-support programs, or other forms of career support could potentially increase the attractiveness of work with older persons, particularly in a context of prolonged economic instability. Such measures would require further evaluation to determine which forms of financial support are feasible and effective in the Lebanese healthcare system.

Second, the association between willingness and having several close non-family relationships with older adults suggests a potential role for sustained interpersonal exposure. Education on the care of older persons may benefit from opportunities for students and trainees to interact with older adults across different settings, including clinical placements, community programs, and intergenerational activities. The present findings should not be interpreted as demonstrating that increasing the number of relationships will necessarily increase willingness, particularly because the quality of contact was not assessed. Nevertheless, they provide a basis for further examining whether sustained and meaningful contact with multiple older adults can contribute to greater interest in work with older persons.

Third, the absence of statistically significant associations in the present sample between willingness and ageism, knowledge about aging, or perceived efficacy of education and training suggests that neither attitudinal interventions nor additional classroom-based education should be considered sufficient on their own to address workforce shortages in the care of older persons. Efforts to prepare healthcare professionals for an aging population may need to combine educational content with direct exposure to older adults and appropriate professional and financial incentives. At the policy level, these measures should be considered alongside broader efforts to strengthen services for older persons and develop a comprehensive national approach to aging and older-adult care in Lebanon.

An additional consideration is that reluctance to work in the care of older persons may not necessarily reflect reluctance to work with older adults themselves. In Lebanon, where nursing homes constitute an important part of the existing formal care structure, some healthcare professionals may be discouraged by the characteristics of the available work settings, including the structure of nursing home-based care, rather than by the older population [10]. This distinction is important because willingness to work with older adults may differ depending on whether care is provided in nursing homes, hospitals, community-based services, home care, or other settings. For instance, working in nursing home settings has often been associated with stress and burnout [81]. Expanding and diversifying care structures for older persons could therefore represent another potential strategy for addressing workforce shortages, alongside financial incentives and opportunities for meaningful contact with older adults. Future research should examine whether reluctance to pursue careers caring for older persons is related to perceptions of older adults, perceptions of specific care settings, or broader concerns about working conditions, organizational structure, workload, and professional opportunities. Such research could help identify whether workforce strategies should focus primarily on changing perceptions of the care of older persons, improving the environments in which such care is delivered, or both.

## 5. Conclusions

This study examined factors associated with willingness to work with older adults among healthcare professionals and students in Lebanon. Financial satisfaction and close relationships with several non-family older adults were positively associated with willingness, whereas no statistically significant associations were observed for ageism, knowledge about aging, or perceived efficacy of education and training. These findings suggest that willingness to work with older adults may be influenced by practical and relational factors that are distinct from general attitudes toward aging or perceived educational preparedness. In the context of Lebanon’s aging population and prolonged healthcare and economic crises, these findings highlight the need to consider financial conditions and meaningful exposure to older adults when addressing the workforce caring for older persons.

Furthermore, educational institutions could strengthen opportunities for sustained intergenerational contact and practical exposure to the care of older persons, while healthcare organizations and policymakers could consider how financial incentives, working conditions, and career development opportunities affect the attractiveness of this field.

Future research should examine these associations using longitudinal and representative samples and should include more comprehensive measures of willingness and financial circumstances. Further studies should also assess the quality and frequency of intergenerational contact, objective exposure to education on the care of older persons and clinical practice, and other career-related factors that may contribute to willingness to work with older adults.

## Figures and Tables

**Table 1:** Sociodemographic and other characteristics of participants (N = 209).

Characteristic	N (%)
**Sex**	
Female	156 (74.6%)
Male	53 (25.4%)
**Education Level**	
Secondary education	36 (17.2%)
Technical education (BT/TS)	15 (7.2%)
Bachelor’s degree (BSc/BA)	102 (48.8%)
Master’s degree (MSc/MA)	35 (16.7%)
Medical degree (MD)	18 (8.6%)
Doctorate (PhD)	3 (1.4%)
**Employment Status**	
Student	116 (55.5%)
Employed	74 (35.4%)
Not currently working	19 (9.1%)
**Years of Education in Elder-Related Field**	
0–1 year	15 (7.2%)
2 years	14 (6.7%)
3 years	76 (36.4%)
More than 3 years	104 (49.8%)
**Work Experience**	
0–1 years	84 (40.2%)
2–3 years	48 (22.9%)
4–5 years	11 (5.3%)
More than 5 years	66 (31.6%)
**Currently Works with Older Adults**	
No	58 (27.8%)
Yes	151 (72.2%)
**Has Taken Courses on Aging/Older Adults**	
No	73 (34.9%)
Yes	136 (65.1%)
**Close Relationship with an Older Person (Non-Family)**	
No	45 (21.5%)
Yes, with many older people	129 (61.7%)
Yes, with one older person	35 (16.7%)
**Elder Family Members**	
No	23 (11.0%)
Yes, more than one older person	149 (71.3%)
Yes, one older person	37 (17.7%)

**Table 2:** Descriptive statistics for study variables (N = 209).

Variable	Mean	SD
Age	28.56	12.01
Willingness to Work with Older Adults	3.11	0.97
Efficacy	2.17	0.86
Financial Satisfaction	3.02	1.07
Ageism	52.32	13.98
Knowledge about Aging	12.74	2.77

**Table 3:** Bivariate analyses of sociodemographic and other variables with willingness scores.

Variable	Mean ± SD	*p*	Effect Size
**Sex**		0.439	−0.067
Female	3.10 ± 0.89		
Male	3.15 ± 1.17		
**Education Level**		0.876	0.000
Secondary education	3.19 ± 1.01		
Technical education	3.20 ± 0.56		
Bachelor’s degree	3.02 ± 0.97		
Master’s degree	3.20 ± 1.08		
Medical degree	3.17 ± 1.04		
Doctorate	3.33 ± 0.58		
**Employment Status**		0.417	0.000
Employed	3.23 ± 0.97		
Not working	3.05 ± 0.62		
Student	3.04 ± 1.01		
**Years of Education in Elder-Related Field**		0.361	0.001
0–1 year	3.33 ± 0.82		
2 years	3.21 ± 1.05		
3 years	2.96 ± 1.03		
More than 3 years	3.17 ± 0.93		
**Work Experience**		0.324	0.002
0–1 years	3.18 ± 0.92		
2–3 years	2.94 ± 1.06		
4–5 years	2.82 ± 1.17		
More than 5 years	3.20 ± 0.92		
**Currently Works with Older Adults**		0.922	0.015
No	3.12 ± 0.99		
Yes	3.11 ± 0.96		
**Has Taken Courses on Aging/Older Adults**		0.877	−0.022
No	3.10 ± 1.02		
Yes	3.12 ± 0.94		
**Close Relationship with Older Person (Non-Family)**		**0.002**	**0.051**
No	2.76 ± 0.91		
Yes, with many older people	3.30 ± 0.92		
Yes, with one older person	2.89 ± 1.05		
**Elder Family Members**		0.141	0.009
No	2.74 ± 1.01		
Yes, more than one older person	3.17 ± 0.93		
Yes, one older person	3.11 ± 1.05		

**Note.** Effect sizes correspond to ω^2^ for ANOVAs, Cohen’s d for independent-samples *t*-tests, and the rank-biserial correlation for the Mann–Whitney U test (used for Sex). Bold *p*-values and effect sizes indicate statistically significant associations (*p* < 0.05).

**Table 4:** Correlation matrix of study variables.

Variable	1	2	3	4	5	6
1. Age	—					
2. Efficacy	0.163	—				
*p*-value	**0.019**	—				
3. Financial Satisfaction	−0.042	0.084	—			
*p*-value	0.549	0.227	—			
4. Ageism	0.049	−0.093	−0.008	—		
*p*-value	0.483	0.179	0.906	—		
5. Knowledge	0.057	0.080	0.011	**−0.225**	—	
*p*-value	0.416	0.247	0.865	**0.001**	—	
6. Willingness	0.073	0.099	**0.202**	0.082	−0.020	—
*p*-value	0.294	0.155	**0.003**	0.235	0.776	—

Bold values indicate statistical significance at the *p* < 0.05 level.

**Table 5:** Ordinal logistic regression (logit link) with Willingness as dependent variable.

Predictor	*B*	SE	Wald χ^2^	*p*	95% CI for B	*OR*	95% CI for OR
**Thresholds**							
Willingness = 1	−0.488	0.832	0.344	0.558	[−2.120, 1.143]	—	—
Willingness = 2	1.010	0.815	1.538	0.215	[−0.586, 2.607]	—	—
**Willingness = 3**	**3.092**	**0.842**	**13.480**	**<0.001**	**[1.442, 4.743]**	**—**	**—**
**Willingness = 4**	**5.345**	**0.900**	**35.259**	**<0.001**	**[3.581, 7.109]**	**—**	**—**
**Location (predictors)**							
Efficacy	0.136	0.153	0.789	0.375	[−0.164, 0.436]	1.146	[0.849, 1.547]
Ageism	0.014	0.009	2.284	0.131	[−0.004, 0.032]	1.014	[0.996, 1.033]
**Financial Satisfaction**	**0.341**	**0.124**	**7.556**	**0.006**	**[0.098, 0.584]**	**1.406**	**[1.103, 1.793]**
**Close Relationship with Older Person (ref.: Yes, with one older person)**							
No	−0.224	0.443	0.255	0.614	[−1.093, 0.645]	0.799	[0.335, 1.906]
**Yes, with many older people**	**0.827**	**0.378**	**4.793**	**0.029**	**[0.087, 1.567]**	**2.286**	**[1.091, 4.792]**
**Elder Family Members (ref.: Yes, one older person)**							
No	−0.305	0.527	0.336	0.562	[−1.338, 0.727]	0.737	[0.262, 2.069]
Yes, more than one older person	−0.200	0.367	0.298	0.585	[−0.919, 0.518]	0.819	[0.399, 1.679]
**Model fit and assumption tests**							
Model fit (LR test)	−2LL intercept only = 563.349; −2LL final = 537.918; χ^2^(7) = 25.431, *p* = 0.001						
Goodness-of-fit	Pearson χ^2^(801) = 933.179, *p* = 0.001; Deviance χ^2^(801) = 533.759, *p* = 1.000						
Pseudo R^2^	Cox and Snell = 0.115; Nagelkerke = 0.123; McFadden = 0.045						
Test of parallel lines	−2LL general = 504.600; χ^2^(21) = 33.318, *p* = 0.043						

**Note.** Only factors that showed a *p* < 0.25 in the bivariate analyses were included in the regression model. *B* = log-odds estimate; SE = standard error; Wald χ^2^ tested on 1 df; CI = confidence interval; OR = odds ratio (exp[B]). Positive B (OR > 1) indicates higher odds of being in a higher Willingness category. Thresholds are the cut-points between adjacent Willingness categories; odds ratios are not applicable to thresholds. Bold values indicate statistically significant associations (*p* < 0.05). Reference categories are indicated in parentheses (redundant parameters set to zero). The test of parallel lines was significant (*p* = 0.043), suggesting the proportional odds assumption may not fully hold.

## Data Availability

The data presented in this study are available on request from the corresponding author due to institutional ethical and legal reasons.

## Supplementary Materials

The following supporting information can be downloaded at https://www.mdpi.com/article/10.3390/healthcare14213584/s1: Supplementary Material S1: Frequency of Professions; Supplementary Material S2: Multiple Linear Regression; Supplementary Material S3: STROBE Statement.
