# Supplementary material for: Factors Associated with Willingness to Work with Older Adults Among Healthcare Professionals and Students: A Cross-Sectional Study in a Polycrisis Context
Source: Healthcare (Basel). 2026 Oct 23;14(21):3584. doi: 10.3390/healthcare14213584 (PMC13762578; doi:10.3390/healthcare14213584)
Supplement: Supplementary file 1 [file healthcare-14-03584-s001.zip › Supplementary Materials S3 STROBE_checklist.pdf]

STROBE Statement—Checklist of items that should be included in reports of ***cross-sectional studies***

	Item No	Recommendation	Reported on Page No.
**Title and abstract**	1	(*a*) Indicate the study's design with a commonly used term in the title or the abstract	p. 1 (title); p. 1 (running title)
		(*b*) Provide in the abstract an informative and balanced summary of what was done and what was found	p. 2 (Abstract)
**Introduction**			
Background/rationale	2	Explain the scientific background and rationale for the investigation being reported	p. 2–5 (Introduction, paras. 1–6)
Objectives	3	State specific objectives, including any prespecified hypotheses	p. 5–6
**Methods**			
Study design	4	Present key elements of study design early in the paper	p. 1; p. 6
Setting	5	Describe the setting, locations, and relevant dates, including periods of recruitment, exposure, follow-up, and data collection	Dates of recruitment/data collection are not stated in the main text. Recruitment method is described on p. 6–7 (online survey via WhatsApp groups); see Supplementary Materials for the full sample description (p. 6).
Participants	6	(*a*) Give the eligibility criteria, and the sources and methods of selection of participants	p. 6
Variables	7	Clearly define all outcomes, exposures, predictors, potential confounders, and effect modifiers. Give diagnostic criteria, if applicable	p. 7–9 (variable definitions in Data Collection)
Data sources/ measurement	8*	For each variable of interest, give sources of data and details of methods of assessment (measurement). Describe comparability of assessment methods if there is more than one group	p. 7–9 (measurement details for each variable)
Bias	9	Describe any efforts to address potential sources of bias	Not explicitly stated as a dedicated bias-mitigation step in Methods. Translation/back-translation and pilot-testing procedures (p. 8–9) address measurement/translation bias; selection and

			social-desirability bias are discussed retrospectively in Limitations (p. 21–22).
Study size	10	Explain how the study size was arrived at	p. 6 (G*Power calculation)
Quantitative variables	11	Explain how quantitative variables were handled in the analyses. If applicable, describe which groupings were chosen and why	p. 9 (normality assessed via skewness/kurtosis). Sociodemographic variables are presented in the categories used in the questionnaire (Table 1, p. 10–11); no separate rationale is given for these groupings.
Statistical methods	12	(*a*) Describe all statistical methods, including those used to control for confounding	p. 9–10
		(*b*) Describe any methods used to examine subgroups and interactions	Not reported (no subgroup or interaction analyses were conducted).
		(*c*) Explain how missing data were addressed	Not reported; missing-data handling is not described. All tables report N = 209 throughout (p. 6; Table 1, p. 10), suggesting a complete-case dataset, but this is not stated explicitly.
		(*d*) If applicable, describe analytical methods taking account of sampling strategy	Not applicable — convenience (non-probability) sample; no weighting or complex design-based methods were used (p. 6).
		(*e*) Describe any sensitivity analyses	p. 16–17 (linear regression reported as a sensitivity analysis); see also p. 9–10 for the justification for switching to ordinal logistic regression.
**Results**			
Participants	13*	(a) Report numbers of individuals at each stage of study—eg numbers potentially eligible, examined for eligibility, confirmed eligible, included in the study, completing follow-up, and analysed	Only the final analytic sample (N = 209) and the minimum required sample size (N = 178)

			are reported (p. 6). Numbers at earlier stages (e.g., surveys opened/started) and reasons for non-participation are not reported.
		(b) Give reasons for non-participation at each stage	Not reported (see response to Item 13a).
		(c) Consider use of a flow diagram	No flow diagram is included.
Descriptive data	14*	(a) Give characteristics of study participants (eg demographic, clinical, social) and information on exposures and potential confounders	Table 1, p. 10–11
		(b) Indicate number of participants with missing data for each variable of interest	Not reported (number of participants with missing data per variable is not indicated).
Outcome data	15*	Report numbers of outcome events or summary measures	p. 12; Table 2, p. 12
Main results	16	(*a*) Give unadjusted estimates and, if applicable, confounder-adjusted estimates and their precision (eg, 95% confidence interval). Make clear which confounders were adjusted for and why they were included	Unadjusted: Table 3 (bivariate analyses), p. 13–14, and Table 4 (correlations), p. 14 — these report p-values and effect sizes/r, but not 95% CIs. Adjusted: Table 5 (ordinal logistic regression), p. 15–16, reporting B, OR, and their 95% CIs.
		(*b*) Report category boundaries when continuous variables were categorized	Not applicable — no continuous variables were categorized for analysis.
		(*c*) If relevant, consider translating estimates of relative risk into absolute risk for a meaningful time period	Not applicable — relative risk was not translated into absolute risk; odds ratios are reported as estimated (Table 5, p. 15–16).
Other analyses	17	Report other analyses done—eg analyses of subgroups and interactions, and sensitivity analyses	p. 16–17 (linear regression sensitivity analysis)
**Discussion**			
Key results	18	Summarise key results with reference to study	p. 17 (opening

		objectives	paragraph of Discussion)
Limitations	19	Discuss limitations of the study, taking into account sources of potential bias or imprecision. Discuss both direction and magnitude of any potential bias	p. 21–23 (Section 4.6, Limitations)
Interpretation	20	Give a cautious overall interpretation of results considering objectives, limitations, multiplicity of analyses, results from similar studies, and other relevant evidence	p. 20–21 (Section 4.5, Attitudes and Willingness to Work with Older Adults)
Generalisability	21	Discuss the generalisability (external validity) of the study results	p. 21–22 (within Limitations: convenience sampling and nursing-weighted sample)
**Other information**			
Funding	22	Give the source of funding and the role of the funders for the present study and, if applicable, for the original study on which the present article is based	p. 25

*Give information separately for exposed and unexposed groups.

Note: An Explanation and Elaboration article discusses each checklist item and gives methodological background and published examples of transparent reporting. The STROBE checklist is best used in conjunction with this article (freely available on the Web sites of PLoS Medicine at http://www.plosmedicine.org/, Annals of Internal Medicine at http://www.annals.org/, and Epidemiology at http://www.epidem.com/). Information on the STROBE Initiative is available at www.strobe-statement.org.
