# Supplementary material for: Factors Associated with Willingness to Work with Older Adults Among Healthcare Professionals and Students: A Cross-Sectional Study in a Polycrisis Context
Source: Healthcare (Basel). 2026 Oct 23;14(21):3584. doi: 10.3390/healthcare14213584 (PMC13762578; doi:10.3390/healthcare14213584)
Supplement: Supplementary file 1 [file healthcare-14-03584-s001.zip › Supplementary Materials S1 Frequency of Professions.pdf]

Table S1. Frequency of Professions

**Profession**	**Frequency**
Nursing	113
Medicine	20
Other healthcare field	19
Clinical Psychology	11
Dentistry	8
Nutrition and Dietetics	6
Occupational Therapy	6
Physiotherapy / Physical Therapy	5
Pharmacy	5
Speech and Language Therapy	4
Medical Laboratory Sciences	4
Public Health	3
Radiology and Medical Imaging	2
Medical Social Work	2
Psychomotor Therapy	1
**Total**	**209**
