# Supplementary material for: Factors Associated with Willingness to Work with Older Adults Among Healthcare Professionals and Students: A Cross-Sectional Study in a Polycrisis Context
Source: Healthcare (Basel). 2026 Oct 23;14(21):3584. doi: 10.3390/healthcare14213584 (PMC13762578; doi:10.3390/healthcare14213584)
Supplement: Supplementary file 1 [file healthcare-14-03584-s001.zip › Supplementary Materials S2 Multiple Linear Regression.pdf]

Supplementary Materials

S2. Sensitivity Analysis: Multiple Linear Regression

A multiple linear regression model was initially planned to examine the factors associated with willingness to work with older adults. Because the diagnostic checks described in Section S2 indicated that the linearity assumption was not adequately met, an ordinal logistic regression was retained as the primary analysis (Table 5 in the main text). The multiple linear regression model is reported here as a sensitivity analysis, using the same set of predictors: efficacy, ageism, financial satisfaction, close relationship with an older person, and elder family members.

The linear model was statistically significant and explained 10.2% of the variance in willingness ($R^2 = 0.102$, Adjusted $R^2 = 0.071$, $p = .003$). Financial satisfaction ($B = 0.150$, $SE = 0.061$, $t = 2.459$, $p = .016$, 95% CI [0.030, 0.270]) and having a close relationship with many older people, relative to having no such relationship ($B = 0.481$, $SE = 0.189$, $t = 2.545$, $p = .011$, 95% CI [0.108, 0.854]), were significant positive predictors. Efficacy, ageism, having a close relationship with only one older person, and having elder family members were not significant. These results are consistent with those of the ordinal logistic regression, in which financial satisfaction and close relationships with many older people were likewise the only significant predictors.

Table S2. Multiple linear regression with Willingness as dependent variable ($R^2 = 0.102$, Adjusted $R^2 = 0.071$, $p = .003$).

Predictor	B	SE	t	p	95% CI for B
**(Constant)**	**1.867**	**0.375**	**4.979**	**< .001**	**[1.128, 2.606]**
Efficacy	0.063	0.077	0.818	0.417	[−0.089, 0.215]
Ageism	0.006	0.005	1.200	0.218	[−0.004, 0.016]
**Financial Satisfaction**	**0.150**	**0.061**	**2.459**	**0.016**	**[0.030, 0.270]**
**Close Relationship with Older Person (ref.: No)**					
Yes, with many older people	**0.481**	**0.189**	**2.545**	**0.011**	**[0.108, 0.854]**
Yes, with one older person	0.085	0.224	0.379	0.703	[−0.357, 0.527]
**Elder Family Members (ref.: No)**					
Yes, more than one older person	0.020	0.241	0.083	0.933	[−0.455, 0.495]

Predictor	B	SE	t	p	95% CI for B
Yes, one older person	0.149	0.266	0.560	0.577	[−0.376, 0.674]

Note. B = unstandardized coefficient; SE = standard error; CI = confidence interval. t values were computed as B/SE and 95% CIs as B ± 1.972 × SE (df = 201) from the reported estimates, so they may differ slightly from software output because of rounding. Bold values indicate statistically significant associations (p < .05). Reference categories are indicated in parentheses.

S3. Assessment of Linear Regression Assumptions

The assumptions of the multiple linear regression model were assessed visually using a plot of residuals against predicted values and a Q-Q plot of standardized residuals (Figure S1).

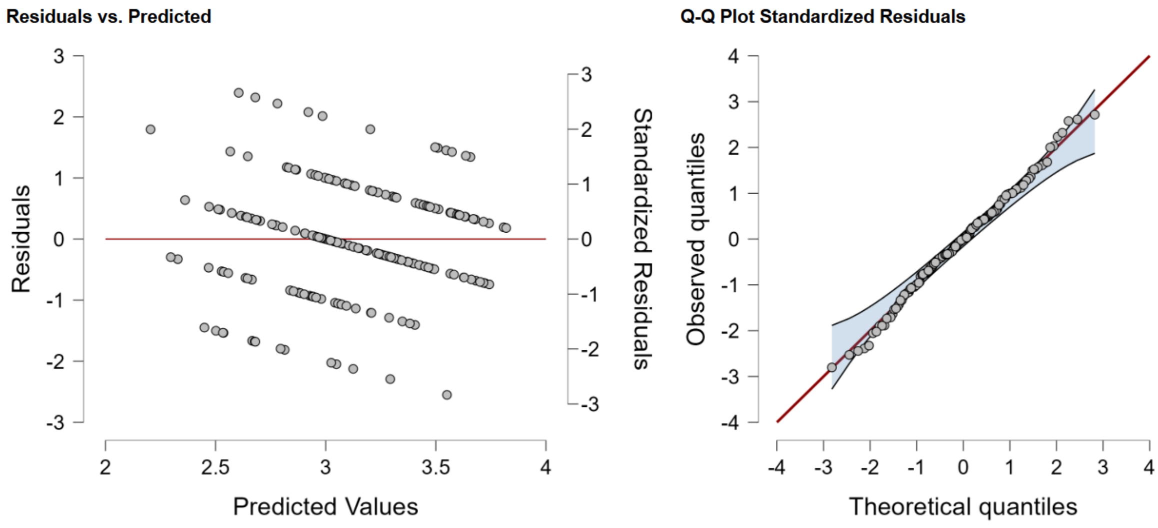

Figure S1. Diagnostic plots for the multiple linear regression model. Left: residuals versus predicted values. Right: Q-Q plot of standardized residuals with 95% confidence band.

Linearity and measurement structure

Assessment: Violated / issues present.

In the residuals versus predicted values plot, the points fall along distinct, parallel diagonal bands sloping downward from left to right, rather than forming a random horizontal cloud around zero. This banded pattern typically arises when the dependent variable is discrete or ordinal, as is the case for willingness, which was measured on a five-point Likert scale. Because the residuals systematically follow these bands, the

assumption of a continuous outcome with linearly modeled, randomly distributed errors is not adequately met, and a linear model is not well suited to this data structure.

Homoscedasticity (constant variance)

Assessment: Satisfied, with caveats.

The vertical spread of the residuals remains fairly consistent across the range of predicted values (approximately 2.0 to 4.0), with residuals falling roughly between −2.5 and +2.5, and there is no pronounced funnel shape in which the spread widens or narrows at either end. However, because the banded structure produced by the discrete outcome compromises the linear model itself, the evaluation of homoscedasticity is secondary to the measurement-structure issue described above.

Normality of residuals

Assessment: Satisfied.

In the Q-Q plot of standardized residuals, the points align closely with the reference line across nearly the entire range of theoretical quantiles (approximately −2.5 to +2.5), and almost all observations remain within the shaded 95% confidence band. The residuals are therefore approximately normally distributed.

Overall conclusion

Although the residuals were approximately normally distributed and showed reasonably constant variance, the clear banded pattern in the residuals versus predicted values plot reflects the ordinal nature of the outcome and indicates that the linear model is not appropriate for these data. An ordinal logistic regression was therefore used as the primary analysis, and the linear regression is presented only as a sensitivity analysis. The consistency of the significant predictors across both models supports the robustness of the main findings.
